# Frequency and predictors of health services use by Native Hawaiians and Pacific Islanders: evidence from the U.S. National Health Interview Survey

**DOI:** 10.1186/s12913-018-3368-3

**Published:** 2018-07-21

**Authors:** Marie-Rachelle Narcisse, Holly Felix, Christopher R. Long, Teresa Hudson, Nalin Payakachat, Zoran Bursac, Pearl A. McElfish

**Affiliations:** 1 Office of Community Health and Research, University of Arkansas for Medical Sciences Northwest, 1125 North College Ave, Fayetteville, AR 72703 USA; 2 Fay W. Boozman College of Public Health, University of Arkansas for Medical Sciences, 4301 West Markham St, Little Rock, AR 72205 USA; 3 Division of Health Services Research, University of Arkansas for Medical Sciences, 4301 West Markham St, Little Rock, AR 72205 USA; 4 Division of Pharmaceutical Evaluation and Policy, University of Arkansas for Medical Sciences, 4301 West Markham St, Little Rock, AR 72205 USA; 5 Division of Biostatistics, University of Tennessee Health Science Center, 910 Madison Ave, Memphis, TN 38163 USA

**Keywords:** Andersen’s model of health services use, Emergency department, outpatient services, Native Hawaiians and other Pacific islanders, National Health Interview Survey, Stereotype logistic regressions

## Abstract

**Background:**

Native Hawaiians and Pacific Islanders (NHPIs) are one of the fasting growing racial groups in the United States (US). NHPIs have a significantly higher disease burden than the US population as a whole, yet they remain underrepresented in research. The purpose of this study is to examine factors associated with health care utilization among NHPIs.

**Methods:**

Drawing from the 2014 NHPI-National Health Interview Survey, we used stereotype logistic regressions to examine utilization of emergency department (ED) and outpatient services among 2172 individuals aged 18 and older.

**Results:**

NHPIs with chronic diseases were twice as likely to be multiple ED users and nearly four times as likely to be frequent-users of outpatient services. Social support played a protective role in preventing multiple use of ED. Having a usual source of care made it more than eight times as likely to be a frequent-user of outpatient services. Use of eHealth information increased the odds of using ED and outpatient services. Ability to afford health care increased the odds of using outpatient services. There was no association between health insurance coverage and use of ED and outpatient services among NHPIs.

**Conclusions:**

This research provides the first available national estimates of health services use by NHPIs. Efforts to improve appropriate use of health services should consider leveraging the protective factors of social support to reduce the odds of frequent ED use, and having a usual source of care to increase use of outpatient services.

## Background

Native Hawaiians and Pacific Islanders (NHPIs) are one of the fasting growing racial groups in the United States (US). Between 2000 and 2010, the NHPI population grew at a rate of 40.1%, and the US has 1.3 million persons reporting their race as NHPI [1, 2]. Despite this rapid growth, NHPI have been underrepresented in research. Much of the population-based research aggregates data on NHPI and Asian Americans, which can obscure disparities between these two heterogeneous subgroups. The limited data available demonstrates that NHPIs have a significantly higher disease burden than Asians Americans, as well as the US population as a whole [3–9]. With the exception of Asian Americans, members of racial and ethnic minority groups are more likely to face barriers to access primary care services [10]. For example, Whites were more likely to use outpatient services than Blacks (315 per 100 persons vs. 229 per 100 persons) [11]. Inability to pay for health care services and lack of a usual source of care affect access to primary care and can lead to poorer health outcomes, including higher mortality rates, lower quality of life, and inappropriate use (underuse, overuse, and misuse) of emergency department (ED) services [12–14]. Results from the 2014 NHIS indicate that 18.6% of American adults aged 18–64 years visited an ED at least one time in the past year and 6.7% visited an ED two or more times [10]. Less is known about the frequency and predictors of health services use by NHPIs living in the US.

### Conceptual framework

Ronald Andersen developed the Behavioral Model of Health Services Use to improve understanding of how health care services were distributed and to support the development of interventions to improve racial and ethnic disparities in access to quality health care [15]. Since its development in 1968, the model has become one of the most applied models to the study of health services use [16–20]. Andersen posited that an individual’s decision to use health services is driven by a variety of factors that includes the individual’s socio-demographic characteristics (predisposing factors), resources the individual has to help facilitate access to services (enabling factors), and whether she/he has a health condition that warrants care (actual or perceived need factors) [15, 21]. A consistent finding in studies based on Andersen’s model of health services use is the importance of “need” variables in explaining utilization variance [22]. Although there is reason to believe that people self-reporting better health status and no chronic conditions would be less frequent users of healthcare services, there is a complex relationship between psychological/subjective/perceived and physiological/objective/evaluated health status [23, 24]. Perceived health status and evaluated disease status are sometimes shown to be closely aligned and other times shown to be discordant [24, 25].

Commonly studied predisposing factors are age, sex, education level, marital status, employment status, language, ethnicity, trust/familiarity with medical organizations, region of residence, and community structure. Enabling factors (e.g. income, health insurance coverage, usual source of care, affordability of health care, social support, health care market, health care safety net supply, health care provider diagnosis) either facilitate or inhibit access to health services. Although availability of health-related information has been explored as an enabling component of Andersen’s model of health services utilization [26], electronic Health (eHealth) information has not been operationalized as part of the model. As patients are increasingly managing their health with the aid of electronic tools, eHealth is becoming a key enabling factor of health services utilization [27–29].

### Research objectives

This study applied Andersen’s model of health services use to examine relationships between predisposing, enabling, and needs factors and health care services utilization among NHPI adults in the US. Specifically, the study examined their independent effects on utilization of ED and outpatient services.

## Methods

### Data source

This cross-sectional study was based on data from the 2014 NHPI-National Health Interview Survey (NHIS), the first population-based study designed exclusively to measure the health of NHPIs in the US. To ensure representation of the NHPI population in the US, the National Center for Health Statistics (NCHS) used stratified multistage area probability sampling. NHPI respondents were randomly selected from families within households to answer a questionnaire based on 2014 NHIS survey. A fuller description of the survey methodology and design can be found elsewhere [11, 30–32].

### Study population

NHPIs are people of cultural-ethnic descent from Hawaii, Guam, Samoa, or other Pacific Islands [27]. In 2014, out of the 2250 NHPI adult (age ≥ 18 years) respondents, 51% self-identified as NHPI alone, and 49% as NPHI in combination with one or more other racial identities. For those who reported NPHI in combination with one or more other races, only adults who reported NHPI as their primary race were included, reducing the analytic sample to 2172 individuals.

### Measures

#### Health care services use

We examined variables associated with health care services utilization from two of the most common settings in the delivery system: ED and outpatient services. For ED services, respondents were asked about the number of times in ER/ED in the past 12 months. Responses were “0; 1; 2-3; 4-5; 6 or more.” We re-classified NHPIs as ED non-users, single-users (1 visit), and multiple-users (≥ 2 visits) based on the frequency of their ED visits in the past 12 months, including visits that led to hospital admissions and those that did not.

For outpatient services, respondents were asked about the total number of office visits in the past 12 months. Responses were: “0; 1; 2-3; 4-5; 6-7; 8-9; 10-12; 13-15; 16 or more.” We re-categorized individuals based on their total number of outpatient visits to a health care professional in the past year as non-users (0), occasional-users [1–3], moderate-users [4–9], and frequent-users (≥ 10 visits) [33–35].

#### Determinants of health care services use

For the predisposing factors, we included age (in years), sex (female/male), education level (less than high-school; high-school/GED; more than high-school), marital status (married, living with a partner/other), employment status (worked within the past 2 weeks, 12 months/not worked within the past 2 weeks, 12 months, never worked), and nativity (born in the US, yes/no).

For the enabling factors, we used questions regarding ability to afford, access to health care, and neighborhood social support, usual place of care, eHealth information, federal poverty level (FPL), and health insurance coverage. For the first three factors, we conducted exploratory factor analysis with a Varimax rotation. A variable was considered related to a factor if it produced a pattern coefficient of 0.55 or better [36].

Ability to afford health care was captured by the following questions: “During the past 12 months, was there any time when you needed any of the following, but didn’t get it because you couldn’t afford it?: prescription medicines, mental health care or counseling, dental care, eyeglasses, see a specialist, follow-up care.” Responses (yes/no) were summed to form a scale with a range from 0 to 6 (α = 0.82).

We created an index to represent barriers to health care access in the past 12 months from the following items: “Did you have any trouble finding a general doctor or provider who would see you?”; “Have you delayed getting care for any of the following reasons because (a) you couldn’t get an appointment soon enough?, (b) you couldn’t get through on the telephone?”; “Were you told by a doctor’s office or clinic that they would not accept you as a new patient?”. Responses (yes/no) were summed to obtain a score ranging from 0 to 4 (α = 0.68).

Social support was conceptualized by items measured on a four point Likert-scale: “People in this neighborhood help each other out; There are people I can count on in this neighborhood; People in this neighborhood can be trusted; and This is a close-knit neighborhood”. Answers “Definitely disagree” to “Definitely agree”, and summed to obtain a score with a range of 1 to 16 (α = 0.93).

Usual place of care was captured by the question: “Is there a place that you usually go to when you are sick or need advice about your health?” (yes/no). Use of eHealth information was defined by the question: “During the past 12 months, did you get information from the internet about your health, medical treatments, or rehabilitation services”? (yes/no). We included federal poverty level (< 100% FPL, 100–199% 200–399%, and ≥ 400% FPL) and health insurance coverage (yes/no).

For need factors, we evaluated the number of chronic diseases (CD = 0,1, ≥2) diagnosed by a health care provider. The list of CDs was derived from the Centers for Disease Control and Prevention and Agency for Healthcare Research and Quality most prevalent CDs [37, 38]. Participants were asked “Have you ever been told by a doctor or other health professional that you had”: diabetes, hypertension, hyperlipidemia, arthritis, cardiovascular diseases (e.g. angina, heart attack, stroke), chronic lung disorders (e.g. asthma, chronic obstructive pulmonary disease, bronchitis, and emphysema), cancer, liver conditions, hepatitis, and mental health illness (e.g. depression, anxiety or other emotional problems). We also included perceived need as the health status of the respondent who self-reported it as being poor, fair, good, very good, or excellent. Since these two operationalizations could show differential outcomes due to specific findings from existing studies, we decided to include both conceptualizations of needs.

### Statistical analyses

We used Stata 14.2 for all statistical analyses [39]. To obtain population estimates representative of NHPI adults, we applied sampling survey weights available in the dataset. To describe populations, we computed weighted means (for continuous variables) and percentages (for categorical variables) along with standard errors and 95% confidence intervals.

To investigate the independent effects of predisposing, enabling, and need factors on the demand for ED and outpatient services, we conducted regression analyses. Because discordance has been observed between patients’ vs. physicians’ ratings of patients’ health, we fitted four separate models: two models for each type of service use, with one regressing service use (either ED or outpatient services) on the predisposing, enabling factors and evaluated needs (CD) and the other using perceived needs (self-reported health status).

We performed stereotype logistic regressions developed by J.A. Anderson [40] in lieu of the proportional-odds model as the “parallel regression” assumption was violated in three out of the four models fitted when we used the Brant test [41], and in lieu of the multinomial logistic regression model as the stereotype logistic model is more parsimonious. Multicollinearity of predictors was assessed for the adjusted models by examining tolerance and variance inflation factor characteristics. Multicollinearity among the predictors was not detected.

All regression analyses were weighted on the basis of the complex NHPI-NHIS sampling survey design.

## Results

### Descriptive analysis

The average NHPI adult respondent was 40.8 years-old, half were females, 49.8% had a high-school education or less, and 33.1% were unemployed. Among NHPIs, 60.7% were married or living in cohabitation, and 70.5% were born in the US.

In 2014, 15.9% of NHPIs lived below the poverty line, whereas 30.2% lived in more affluent households (≥400% FPL), 6.2% were uninsured, and 87.4% had a usual source of routine or preventive care.

Only 19.3% of NHPI adults reported using the internet to obtain health information for their health needs. Inability to afford health care was on average low, with a mean of 0.3 on a scale of 0–6 (se: 0.02), neighborhood social support was on average high, with a mean of 12.4 on a scale of 1–16 (se: 3.1), and barriers to health care access was also low, with a mean of 0.1 on a scale of 0–4 (se: 0.02).

Among NHPIs, 23.9% reported having been diagnosed with one CD, and 36.4% reported having been diagnosed with at least two CDs. Although 86.3% described their health status as good, very good, or excellent, 13.7% rated their health as poor or fair.

While 81.6% of NHPIs did not use the ED at all the past 12 months, 11.4% and 7.0% of NHPIs were single-users and multiple-users, respectively. A little more than a quarter (26.6%) of NHPIs reported not using outpatient services in the past 12 months, 46.1% of NHPIs reported occasional use 1–3 visits per year, and 10.4% of NHPIs frequently visited outpatient services (i.e., ≥10 visits/year). See Table 1.

**Table 1:** Describing NHPIs’ Health Services Use based on Andersen’s Model

*n* = 2172	Percentages or Means^a^	95% Confidence Intervals or standard errors
Health care utilization: Outcomes		
ED/ER visits		
Non-Users	816	791–839
Single-users (1 visit/yr)	114	96–135
Multiple-users (≥2 visits/yr)	70	59–84
Outpatient services		
Non-Users	266	240–293
Occasional-users (1–3 visits/yr)	461	428–494
Moderate-users (4–9 visits/yr)	170	149–193
Frequent-users (≥10 visits/yr)	104	85–126
Predisposing factors		
Age (mean)	408	399–417
Females	502	462–542
Education level		
Less than high school	102	80–130
High-school/GED	396	361–433
High-school or more	502	470–538
Married; living with a partner (yes)	607	575–639
Unemployed	331	285–380
Born in the US (yes)	705	669–739
Enabling factors		
Poverty level		
< 100% FPL	159	129–195
100–199% FPL	241	214–271
200–399% FPL	298	265–333
≥ 400% FPL	302	283–322
Uninsured (yes)	62	45–80
Usual source of routine/preventive care (yes)	874	849–896
Use eHealth information (yes)	193	170–219
Ability to afford health care (mean, standard error)	0.3	0.02
Neighborhood social support (mean, standard error)	12.4	3.1
Barriers to health care access (mean, standard error)	0.1	0.02
Needs		
Evaluated needs (number of CDs)		
0	397	359–437
1	239	213–267
≥ 2	364	339–389
Perceived needs (Health Status)		
Poor-Fair	137	123–151
Good	283	251–317
Very good	300	268–333
Excellent	280	239–326

Source: NHPI-NHIS2014

^a^Weighted means and percentages. Valid estimates do not account for missing data

### Regression analyses

#### Model 1

##### Predicting use of ED services using predisposing, enabling, and evaluated need factors

Results from the stereotype logistic regression analyses indicated there were no predisposing, enabling, or need factors that predicted single use of the ED compared to non-use or multiple use compared to single use. However, several factors predicted multiple use compared to non-use. NHPIs with incomes 100–199% of FPL were significantly more likely to be multiple users than non-users (OR:1.593; CI:1.023–2.482) as compared to those with incomes ≥400% FPL. NHPIs who used eHealth information were more likely to be multiple users than non-users (OR:1.719; CI:1.080–2.736). People with multiple CDs were more than twice as likely to be multiple users than non-users (OR:2.267; CI:1.399–3.672). In contrast, we found that people with stronger social support were more likely to be non-users than multiple users (OR:0.937; CI:0.881;0.997). See Table 2.

**Table 2:** Predicting use of ED services among NHPIs: Odds-Ratio and Confidence Interval (95% CI)^a^

	Model 1: Predisposing, Enabling, and Evaluated Needs				Model 2: Predisposing, Enabling, and Perceived Needs		
	Single vs. Non-Users	Multiple vs. Non-Users	Multiple vs. Single		Single vs. Non-Users	Multiple vs. Non-Users	Multiple vs. Single
Predisposing factors							
Age	1.007 (0.997-1.018)	1.010 (0.993-1.027)	1.002 (0.993-1.011)		1.008 (0.997-1.020)	1.015^*^ (1.001-1.030)	1.007 (0.998-1.015)
Females	0.861 (0.627-1.181)	0.823 (0.538-1.261)	0.957 (0.801-1.143)		0.914 (0.707-1.182)	0.852 (0.526-1.383)	0.933 (0.731-1.191)
High-school or less	1.083 (0.564-2.081)	1.109 (0.487-2.526)	1.024 (0.851-1.232)		1.088 (0.621-1.906)	1.161 (0.451-2.988)	1.067 (0.721-1.581)
High-school/GED	1.208 (0.795-1.835)	1.277 (0.815-2.000)	1.057 (0.901-1.240)		1.088 (0.621-1.906)	1.111 (0.590-2.092)	1.067 (0.721-1.581)
Married/living with partner	0.721 (0.423-1.228)	0.655 (0.333-1.288)	0.908 (0.648-1.273)		0.757 (0.441-1.298)	0.609 (0.279-1.329)	0.806 (0.566-1.148)
Unemployed	1.002 (0.652-1.540)	1.003 (0.575-1.748)	1.001 (0.882-1.135)		0.951 (0.694-1.304)	0.915 (0.518-1.616)	0.962 (0.742-1.246)
Born in the US	1.302 (0.841-2.014)	1.407 (0.810-2.445)	1.081 (0.823-1.420)		1.184 (0.793-1.767)	1.349 (0.703-2.590)	1.140 (0.841-1.544)
Enabling factors							
< 100% FPL	1.387 (0.705-2.731)	1.528 (0.563-4.148)	1.101 (0.716-1.695)		1.158 (0.620-2.164)	1.297 (0.391-4.304)	1.120 (0.621-2.022)
100-199% FPL	1.433 (0.875-2.348)	1.593^*^ (1.023-2.482)	1.112 (0.829-1.492)		1.128 (0.738-1.726)	1.239 (0.633-2.427)	1.098 (0.838-1.440)
200-399% FPL	1.003 (0.656-1.533)	1.004 (0.580-1.738)	1.001 (0.883-1.134)		0.925 (0.645-1.328)	0.871 (0.460-1.650)	0.942 (0.708-1.254)
Uninsured	0.920 (0.263-3.224)	0.898 (0.166-4.850)	0.976 (0.631-1.509)		0.936 (0.369-2.379)	0.890 (0.159-4.982)	0.950 (0.431-2.097)
Usual source of care	1.102 (0.425-2.859)	1.134 (0.315-4.085)	1.029 (0.734-1.443)		1.086 (0.464-2.538)	1.157 (0.251-5.329)	1.066 (0.538-2.110)
Use eHealth information	1.520 (0.917-2.519)	1.719^*^ (1.080-2.736)	1.131 (0.792-1.616)		1.436 (0.912-2.262)	1.901^*^ (1.137-3.180)	1.117 (0.873-1.430)
Ability to afford health care	1.236 (0.990-1.543)	1.316 (0.823-2.105)	1.064 (0.809-1.400)		1.154 (0.980-1.359)	1.290 (0.893-1.864)	1.12 (0.873-1.437)
Neighborhood social support	0.951 (0.884-1.023)	0.937^*^ (0.881-0.997)	0.985 (0.948-1.025)		0.971 (0.914-1.031)	0.948 (0.877-1.026)	0.977 (0.949-1.006)
Barriers to health care access	1.138 (0.809-1.601)	1.183 (0.797-1.756)	1.039 (0.916-1.179)		1.151 (0.837-1.582)	1.283 (0.804-2.048)	1.115 (0.916-1.357)
Evaluated needs (CDs)				Perceived needs (health status)			
1	1.191 (0.763-1.860)	1.254 (0.715-2.199)	1.053 (0.862-1.286)	Good	0.484^*^ (0.282-0.830)	0.275^**^ (0.107-0.708)	0.570 (0.241-1.347)
≥ 2	1.881 (0.971-3.645)	2.267^**^ (1.399-3.672)	1.205 (0.714-2.034)	Very good	0.369^**^ (0.202-0.674)	0.170^***^ (0.074-0.391)	0.462 (0.161-1.324)
				Excellent	0.451^**^ (0.254-0.801)	0.243^**^ (0.090-0.659)	0.540 (0.212-1.374)

Source: NHPI-NHIS 2014

^*^p < 0.05; ^**^p < 0.01; ^***^p < 0.001

^a^Referent: More than high-school; other marital status (widowed, divorced or separated, never married); ≥400% FPL; insured; no usual source of care; did not use eHealth; No CD; poor-fair health)

#### Model 2

##### Predicting use of ED services using predisposing, enabling, and perceived need factors

For each-one unit increase in age, the odds of being a multiple user vs. a non-user increased by 1.015 (CI:1.001–1.030), whereas this association was not found in the other comparison groups. Use of eHealth information was also found to be a significant predictor of multiple vs. non-use of ED services. As reports of eHealth information increased, the odds of an NHPI adult being a multiple-user compared to a non-user nearly doubled (OR:1.901; CI:1.137–3.180).

NHPIs in good health (OR:0.448; CI:0.282–0.830), very good health (OR:0.369; CI:0.202–0.674), or excellent health (OR:0.451; CI:0.254–0.801) as compared to those in poor or fair health were less likely to be single ED users than non-users. Similarly, individuals in good health (OR:0.275; CI:0.107–0.708), very good health (OR:0.170; CI:0.074–0.391), or excellent health (OR:0.243; CI:0.090–0.659) were less likely to be multiple users than non-users. See Table 2.

#### Model 3

##### Predicting use of outpatient services using predisposing, enabling and evaluated need factors

Age was associated with increased use of outpatient services: For each year increase in age, there was a significant increase in the odds that NHPIs would be occasional users vs. non-users (OR:1.090; CI:1.001–1.016), moderate-users vs. non-users (OR:1.019, CI:1.003–1.0035), and frequent-users vs. non-users (OR:1.022; CI:1.005–1.0038), respectively. Being female was positively associated with use of outpatient services: the odds were not only greater for women, but they were also larger as recurrent medical service use increased (OR:1.321; CI:1.001–1.786), (OR:1.852; CI:1.062–3.233), and (OR:2.023; CI:1.124–3.640) for occasional, moderate, and frequent-users, respectively.

Unemployed NHPIs were more likely to be occasional users of outpatient services (OR: 1.421; CI:1.045–1.933), moderate users (OR: 2.177; CI: 1.250–3.791), or frequent-users (OR:2.433; CI:1.303–4.542) than non-users.

Whereas place of birth was not associated with ED use, NHPIs who were born in the US were twice as likely to be moderate-users or frequent-users of outpatient services than non-users as compared to NHPIs born elsewhere (OR:2.177; CI:1.250–3.791) and (OR:2.433; CI:1.303–4.542). Having a usual source of care was found to be a significant enabling predictor of use of outpatient services, making it more than twice as likely that an NHPI adult would be an occasional user (OR:2.335; CI:1.662–3.281), more than six times as likely to be a moderate-user (OR:6.540; CI:3.050–14.027), and more than eight times as likely to be a frequent-user (OR:8.555; CI:3.461–2.869) rather than a non-user. See Table 3.

**Table 3:** Predicting use of outpatient services among NHPIs living in the US: Odds-Ratio and Confidence Interval (95% CI)^a^

	Model 3: Predisposing, Enabling, and Evaluated Needs				Model 4: Predisposing, Enabling, and Perceived Needs		
	Occasional vs. Non-Users	Moderate vs. Non-Users	Frequent vs. Non-Users		Occasional vs. Non-Users	Moderate vs. Non-Users	Frequent vs. Non-Users
Predisposing factors							
Age	1.009^*^ (1.001-1.016)	1.019^*^ (1.003-1.035)	1.022^*^ (1.005-1.038)		1.011^*^ (1.002-1.020)	1.023^*^ (1.005-1.041)	1.028^**^ (1.008-1.049)
Females	1.321^*^ (1.001-1.786)	1.852^*^ (1.061-3.233)	2.023^*^ (1.124-3.640)		1.303^*^ (1.000-1.713)	1.760^*^ (1.067-2.902)	2.001^*^ (1.094-3.659)
High-school or less	1.045 (0.778-1.402)	1.101 (0.563-2.156)	1.117 (0.516-2.417)		1.092 (0.827-1.442)	1.206 (0.645-2.255)	1.259 (0.583-2.718)
High-school/GED	1.015 (0.851-1.211)	1.034 (0.699-1.530)	1.039 (0.664-1.626)		0.989 (0.822-1.189)	0.976 (0.658-1.448)	0.971 (0.598-1.576)
Married/living with partner (yes)	1.171 (0.876-1.564)	1.417 (0.777-2.583)	1.490 (0.744-2.983)		1.127 (0.843-1.506)	1.290 (0.696-2.390)	1.366 (0.646-2.892)
Unemployed	1.421^*^ (1.045-1.933)	2.177^**^ (1.250-3.791)	2.433^**^ (1.303-4.542)		1.393^*^ (1.022-1.899)	2.028^*^ (1.183-3.477)	2.381^*^ (1.218-4.653)
Born in the US (yes)	1.347^**^ (1.089-1.667)	1.935^**^ (1.192-3.140)	2.127^*^ (1.179-3.836)		1.317^*^ (1.062-1.632)	1.798^*^ (1.078-2.999)	2.054^*^ (1.084-3.895)
Enabling factors							
< 100% FPL	0.684 (0.404-1.159)	0.431 (0.160-1.164)	0.382 (0.133-1.101)		0.582 (0.325-1.044)	0.315^*^ (0.122-0.814)	0.242^*^ (0.079-0.741)
100-199% FPL	0.830 (0.609-1.133)	0.663 (0.346-1.270)	0.625 (0.293-1.333)		0.736 (0.537-1.007)	0.519^*^ (0.281-0.958)	0.448^*^ (0.209-0.956)
200-399% FPL	0.766 (0.571-1.028)	0.555^*^ (0.331-0.929)	0.510^*^ (0.286-0.909)		0.719^*^ (0.532-0.971)	0.494^**^ (0.303-0.807)	0.421^**^ (0.234-0.759)
Uninsured	0.694 (0.343-1.404)	0.446 (0.108-1.842)	0.397 (0.076-2.064)		0.734 (0.385-1.399)	0.516 (0.133-2.001)	0.444 (0.085-2.328)
Usual source of care	2.335^***^ (1.662-3.281)	6.540^***^ (3.050-14.027)	8.555^***^ (3.461-21.151)		2.361^***^ (1.603-3.479)	6.255^***^ (3.111-12.578)	9.485^***^ (3.950-22.775)
Use eHealth information	1.517^**^ (1.187-1.938)	2.515^***^ (1.509-4.192)	2.869^**^ (1.580-5.212)		1.589^**^ (1.213-2.081)	2.686^***^ (1.624-4.442)	3.361^***^ (1.887-5.987)
Ability to afford health care	1.214^*^ (1.022-1.442)	1.536^**^ (1.128-2.091)	1.633^*^ (1.126-2.370)		1.185^*^ (1.026-1.369)	1.436^*^ (1.097-1.880)	1.559^**^ (1.135-2.140)
Neighborhood social support	0.980 (0.950-1.012)	0.957 (0.889-1.030)	0.951 (0.873-1.036)		0.984 (0.952-1.017)	0.966 (0.897-1.041)	0.959 (0.875-1.051)
Barriers to health care access	1.043 (0.810-1.342)	1.097 (0.636-1.892)	1.112 (0.599-2.062)		1.087 (0.857-1.379)	1.195 (0.743-1.923)	1.245 (0.699-2.217)
Evaluated needs (CDs)				Perceived needs (health status)			
1	1.253 (0.955-1.643)	1.647 (0.873-3.109)	1.769 (0.872-3.589)	Good	0.533^**^ (0.357-0.796)	0.261^***^ (0.132-0.515)	0.192^***^ (0.078-0.478)
≥ 2	1.716^**^ (1.223-2.407)	3.306^***^ (1.792-6.099)	3.922^***^ (1.979-7.775)	Very good	0.459^**^ (0.281-0.749)	0.190^***^ (0.100-0.361)	0.130^***^ (0.056-0.303)
				Excellent	0.404^**^ (0.235-0.694)	0.145^***^ (0.064-0.329)	0.093^***^ (0.030-0.288)

Source: NHPI-NHIS 2014

^*^p < 0.05; ^**^p < 0.01; ^***^ *p* < 0.001

^a^Referent: More than high-school; other marital status (widowed, divorced or separated, never married); ≥400% FPL; insured; no usual source of care; did not use eHealth; No CD; poor-fair health)

Compared to NHPIs who did not use eHealth information, those who did were more likely to use outpatient services. The odds increased for occasional, moderate, and frequent-users by 1.517 (CI:1.187–1.938), 2.515 (1.509–4.192), and 2.869 (1.509–5.192) times vs. non users, respectively. Ability to afford health care was positively associated with utilization of outpatient services across all three comparison groups as well (OR:1.214; CI:1.022–1.442) (OR:1.536; CI:1.128–2.091), and (OR:1.633; CI:1.126–2.370).

With regard to evaluated needs, relative to their healthier peers, NHPIs with multiple chronic conditions were at greater odds of being occasional, moderate, and frequent-users than non-users (OR:1.761; CI:1.223–2.407); (OR:3.306; CI:1.792–6.099), and (OR:3.922; CI:1.979–7.775). See Table 3.

#### Model 4

##### Predicting use of outpatient services using predisposing, enabling and perceived need factors

Age was positively associated with use of outpatient services by occasional, moderate, and frequent-users vs. non-users: (OR:1.011; CI:1.002–1.02), (OR:1.023; CI:1.005–1.041), and (OR:1.028; CI:1.008–1.049), respectively. Being female was positively associated with use of outpatient services: (OR:1.303; CI:1.000–1.713), (OR:1.761; CI:1.067–2.902), and (OR:2.001; CI:1.094–3.659) for occasional, moderate, and frequent-users vs. non-users. Being unemployed was associated with use of outpatient services: the odds of being a moderate-user or a frequent-user doubled when NHPIs were unemployed (OR:2.028; CI:1.183–3.477) and (OR:2.381; CI:1.218–4.653).

Level of poverty was a predictive enabler of outpatient services use in the NHPI population. As compared to NHPIs living ≥400% FPL, NHPIs living below poverty line were less likely to be moderate-users or a frequent-user: (OR:0.315; CI:0.122–0.814) and (OR:0.242; CI:0.0.079–0.741). Although health insurance coverage was not associated with NHPIs’ use of outpatient services, having a usual source of care was strongly associated with use; the odds across all three comparisons increased by 2.361 (CI:1.603–3.479), 6.255 (3.111–12.578), and 9.485 (CI:3.950–22.775), respectively.

NHPIs who used eHealth were at increased odds of using outpatient services. The odds increased significantly across all user comparisons, with NHPIs who used eHealth information being 3.361 times more likely to frequently use outpatient services (CI:1.887–5.987), 2.686 times more likely to moderately use outpatient services (CI:1.624–4.442), and 1.586 times more likely to occasionally use outpatient services (CI:1.213–2.081) as compared to people who did not use eHealth information.

Across all three categories of users, negative associations were found between perceived health and outpatient services: NHPIs in good health (OR:0.261; CI:0.132–0.515), very good health (OR:0.190; CI:0.100–0.361), or excellent health (OR:0.145; CI:0.064–0.329), as compared to those in poor or fair health were less likely to be moderate-users than non-users. Similarly, individuals in good health (OR:0.192; CI:0.078–0.478), very good health (OR:0.130; CI:0.056–0.303), or excellent health (OR:0.093; CI:0.030–0.288) were less likely to be multiple users than non-users. See Table 3.

## Discussion

This is the first ever national report of health services use by NHPIs residing in the US. In this study, almost half (46.1%) were occasional users (1–3 visits per year) of outpatient services. The study reviewed also that 18.4% NHPIs made a visit to an ED within the last year, which is comparable to the US population as a whole – 18.6% of adult Americans, regardless of race or ethnicity, visited an ED in the last year – [42]; 7.0% were multiple ED users as compared to 6.7% for the overall US population (6.7%) [10]. This suggests that although NHPIs have consistently faced a disproportionate burden of chronic diseases compared with other demographic groups [43], this burden does not necessarily translate into significantly more use of health services.

In this study, we identified a number of determinants of outpatient and ED services. Age was a significant predictor of NHPIs’ frequent use of ED services (when using a measure of perceived need) and frequent use of outpatient services, regardless of the measure of need. This is consistent with a number of studies which have also demonstrated these findings in other populations [44, 45]. Female sex was a predictor of more frequent use of outpatient services. This finding is consistent with other research on the association between sex and outpatient services that has been conducted on samples of community-dwelling and low-income persons in the US [46, 47]. Place of birth was also found to be a significant predisposing predictor for NHPIs to use outpatient services frequently. Those born in the US were significantly more likely to be frequent-users of outpatient services compared to those not born in the US. Studies have found that immigrant adults use less health care than US born [48]. NHPIs who reported not having a job were significantly more likely to report frequent outpatient services, but job status had no effect on ED service use. This is a surprising finding given the evidence that limited financial resources, which presumably an unemployed person would have, is a barrier to accessing health care [49, 50]. However, since in the study population 25.8% and 22.6% of unemployed NHPIs were covered by Medicare and Medicaid, and 19.1% of them were also retired, their status would allow them more time to seek care.

In terms of enabling resources, use of eHealth information by NHPIs was a significant predictor of multiple use of both ED and outpatient services. eHealth refers to “the use of emerging information and communication technology, especially the Internet, to improve or enable health and health care.” [51] The literature is mixed on the effect of eHealth information. Research based on NHIS data has shown that individuals experiencing trouble accessing health care services for reasons unrelated to having health insurance coverage were more likely to report using eHealth information [52]. Our finding is consistent with a study of more than 7000 internet users, which found that those who used eHealth information were more likely to increase health care use (OR:2.9, CI:1.3–6.3, *p* < 0.01) compared to those who did not use it at all [53]. Another study found that those who sought eHealth information used the eHealth information because it was free or because health care was expensive were 90% less likely to use health care services [54]. More research is needed to understand how characteristics of the eHealth user (such as insurance status or income level) affects level and type of health services use.

Among NHPIs, having a usual source of care and being able to pay for health care services were associated with outpatient services but not ED services. This is consistent with other literature that shows these enabling factors predict use of primary care services in other populations [55, 56]. NHPIs with incomes below the FPL were significantly more likely to be frequent-users of ED services than those at the highest income levels. It is possible that those with very low incomes lacked access to primary care services and used the ED as a safety net provider. Studies have shown that ED patients have a high prevalence of material needs such as poverty, housing and food, difficulty paying for health care, and difficulty affording basic expenses [57].

The availability of social support was also a significant factor in predicting multiple ED visits. When social support was high, NHPIs were less likely to be multiple users of emergency services. Given the collectivistic nature of the NHPI culture, this finding is consistent with prior research that shows persons in collectivist cultures often turn to the families and the community for care and only seek out formal health care for emergencies [58].

Many studies have assessed the relationship between need for health care services and use of health care services, finding significant associations between high level of need and high use of services [59]. We also found that NHPIs with a need for health care services were more likely to frequently use health care services, whether need was assessed by number of chronic conditions or by self-reported health status. The results of separate regression analyses for ED and outpatient services use did not show much difference in the significance or magnitude of estimates when the models with perceived needs (self-reported health status) and evaluated needs (health care provider-diagnosed chronic diseases) were fitted. Nevertheless, in models predicting ED services use, older age emerged as a significant predisposing factor even when the influence of perceived needs was held constant. Older age was also associated with more outpatient services use when perceived or evaluated needs were held fixed. The literature supports the observation that older adults use more services overall [60]. One implication of this finding regarding age is the necessity for future research to explore the appropriateness of use of outpatient services as well as ED services by older NHPI adults. While aging is associated with greater medical needs, further study is needed to determine the extent to which effective and efficient health care services use by older NHPI adults is more associated with their evaluated needs rather than perceived needs, or vice versa.

As for poverty levels, lower income was associated with increased use of ED services in the evaluated needs model and decreased use of outpatient services in both evaluated and perceived needs models. This finding suggests that NHPI with low incomes might minimize their outpatient services used due to perceived financial barriers, which can lead to increased ED use – a finding that has been observed in other populations [61]. Research has indeed shown that hospital care is viewed by low socioeconomic individuals as more affordable than ambulatory care [61]. This result suggests that NHPI could benefit from improved linkages to resources to pay for outpatient health services. Furthermore, neighborhood social support was negatively associated with multiple ED services use only in models that controlled for the influence of evaluated needs. Previous research in other populations has suggested community supportive health care services as a form of enabling factor that would prevent ED use [62]. More research is needed to investigate whether NHPI proactively use social support within their community as supportive care services to alleviate chronic diseases and prevent some or much ED use. Unexpectedly, our study did not show any statistically significant associations between having health insurance and use of ED and outpatient services. This is in contrast to other research which shows associations between health insurance coverage and utilization of ED and outpatient services [63–65]. However, we did observe an association between income and ability to afford health care and use of outpatient services which suggests that health insurance coverage may not be an enabling factor in predicting health services use in our study population, but rather the ability to afford health care services. Future research on whether complementary insurance increases the use of ED and outpatient services in the NPHI population is warranted.

### New contributions and limitations

Although this study provides previously unavailable estimates of health service use by NHPIs in the US, and identifies significant predictors of health services use in the NHPI population, the findings must be considered in light of some limitations. First, our model did not consider the roles health care resources (e.g. labor and capital available to deliver health care) and organization (how the delivery system uses its resources) play in individuals’ health care seeking behavior. Second, the publically available data from the NHIS-NHPI does not include zip codes, US state of residence, and rural/urban classification; as such, it was not possible to include health care system characteristics for NHPI respondents’ home communities in the analysis.

Third, the cross-sectional nature of the study makes it difficult to assess the temporal relationship between an exposure or factor (cause: having a usual source of health care) and an outcome (effect: frequency of health services use). It is possible that NHPIs who use health care services frequently may have developed a usual source of health care because of their service use, rather than having a usual source of health care leading to more frequent use of health services. Nevertheless, this study illustrates the frequency of health services use among NHPI and the extent to which they report having a usual source of care (or not); this study’s findings fill a gap in the literature on this subject and can inform future public health action and research. Furthermore, the factors that lead individuals to use of health services are complex, and can have a bidirectional influence. The methods applied in this study did not allow for an examination of these complexities, rather it allowed for a description of individual predictors of service use and the associations they may have with ED and outpatient services. Future studies should consider application of other statistical methods (e.g. path analysis) and longitudinal design which can provide insights into the long-term causal effects of these predictors on ED and outpatient services as well as potential feedback loops.

Fourth, the data on health, disease burden, and health care services utilization available from the NHPI-NHIS is self-reported, and as such, may be affected by recall and/or courtesy bias (e.g. respondents reporting what they believe the interview wants to hear such as indicating their health status is good when it may be poor). However, the NHIS, on which the NHPI-NHIS was based, is the principle survey for collecting information on the health of the US population and is subject to the same limitation.

Fifth, Asians and NHPIs have historically been aggregated in health research, which likely obscured important differences between these two populations. Although our study was based on data that disaggregated Asians from NHPI, the NHPI population is made up of diverse sub-populations, and it was not possible to examine heterogeneity among different sub-populations of NHPI in this study. Despite these limitations, this research provides several new contributions to research. This study is the first available national estimates of health services use by NHPIs. Although availability of health-related information has been studied as an enabling factor, no studies have explored the influence of eHealth information on health care utilization with the Andersen’s model of Health service utilization. To our knowledge, this is also the first study that has used stereotype logistic regression to empirically apply Andersen’s behavioral model of health services use.

## Conclusions

In conclusion, based on the Andersen’s model of health services use, we found that more predisposing and enabling factors were significantly associated with use of outpatient services than with use of ED services. In models which included the evaluated needs of NHPI, low poverty level (100–199% FPL), and use of eHealth information were positively associated with multiple use of ED services whereas increased neighborhood social support was negatively associated with multiple use of ED services vs. no-use. In models which included NHPI perceived needs, only age and use of eHealth information were positively associated with multiple use of ED services. In predicting use of outpatient services among NHPI, we found that age, being female, being unemployed, being born in the US, having a usual source of care, using eHealth information, and being able to afford health care, were all positively associated with outpatient services (occasional, moderate, frequent use vs. no-use) across all four models. Overall, having perceived better health was negatively associated with both use of ED and outpatient services. In contrast, having multiple chronic diseases was positively associated with use of ED and outpatient services.

Previous reports have indicated that NHPIs have poorer health status and disproportionately higher rates of chronic health conditions such as diabetes, obesity and asthma. However, information about NHPIs’ access to health services and actual health services use at the national level has been lacking until now. Knowledge about the determinants of health services utilization can inform health care organizations and policy makers who seek encourage the appropriate use of health care services. Facilitating access to health care and appropriate use of health care services may help address disparities in health status. Efforts to improve appropriate use of health services should consider leveraging the protective factors of social support to reduce the odd of frequent ED use, and promoting having a usual source of care to increase use of outpatient services.

## Abbreviations

CD: Chronic diseases
CI: Confidence interval
ED: Emergency department
ER: Emergency room
FPL: Federal poverty level
NCHS: National Center for Health Statistics
NHIS: National Health Interview Survey
NHPI: Native Hawaiians and Pacific Islanders
OR: Odds-Ratios
