# Correction to: Frequency and predictors of health services use by native Hawaiians and Pacific islanders: evidence from the U.S. National Health Interview Survey

**DOI:** 10.1186/s12913-018-3754-x

**Published:** 2018-12-04

**Authors:** Marie-Rachelle Narcisse, Holly Felix, Christopher R. Long, Teresa Hudson, Nalin Payakachat, Zoran Bursac, Pearl A. McElfish

**Affiliations:** 1 Office of Community Health and Research, University of Arkansas for Medical Sciences Northwest, 1125 North College Ave, Fayetteville, AR 72703 USA; 2 Fay W. Boozman College of Public Health, University of Arkansas for Medical Sciences, 4301 West Markham St, Little Rock, AR 72205 USA; 3 Division of Health Services Research, University of Arkansas for Medical Sciences, 4301 West Markham St, Little Rock, AR 72205 USA; 4 Division of Pharmaceutical Evaluation and Policy, University of Arkansas for Medical Sciences, 4301 West Markham St, Little Rock, AR 72205 USA; 5 Division of Biostatistics, University of Tennessee Health Science Center, 910 Madison Ave, Memphis, TN 38163 USA

**Correction to: BMC Health Services Research (2018) 18:575**

**https://doi.org/10.1186/s12913-018-3368-3**

Following publication of the original article [1], the author reported the following errors in Table 1 and Table 2.

**Table 1:** Describing NHPIs’ Health Services Use based on Andersen’s Model

*n* = 2172	Percentages or Means^a^	95% Confidence Intervals or Standard Errors
Health care utilization: Outcomes		
ED/ER visits		
Non-Users	81.6	79.1–83.9
Single-users (1 visit/year)	11.4	9.6–13.5
Multiple-users (≥2 visits/year)	7.0	5.9–8.4
Outpatient services		
Non-Users	26.6	24.0–29.3
Occasional-users (1–3 visits/year)	46.1	42.8–49.4
Moderate-users (4–9 visits/year)	17.0	14.9–19.3
Frequent-users (≥10 visits/year)	10.4	8.5–12.6
Predisposing factors		
Age (mean)	40.8	39.9–41.7
Females	50.2	46.2–54.2
Education level		
Less than high school	10.2	8.0–13.0
High-school/GED	39.6	36.1–43.3
High-school or more	50.2	47.0–53.8
Married; living with a partner (yes)	60.7	57.5–63.9
Unemployed	33.1	28.5–38.0
Born in the US (yes)	70.5	66.9–73.9
Enabling factors		
Poverty level		
< 100% FPL	15.9	12.9–19.5
100–199% FPL	24.1	21.4–27.1
200–399% FPL	29.8	26.5–33.3
≥ 400% FPL	30.2	28.3–32.2
Uninsured (yes)	6.2	4.8–8.0
Usual source of routine/preventive care (yes)	87.4	84.9–89.6
Use eHealth information (yes)	19.3	17.0–21.9
Ability to afford health care (mean, standard error)	0.3	0.02
Neighborhood social support (mean, standard error)	12.4	3.1
Barriers to health care access (mean, standard error)	0.1	0.02
Needs		
Evaluated needs (number of CDs)		
0	39.7	35.9–43.7
1	23.9	21.3–26.7
≥ 2	36.4	33.9–38.9
Perceived needs (Health Status)		
Poor-Fair	13.7	12.3–15.1
Good	28.3	25.1–31.7
Very good	30.0	26.8–33.3
Excellent	28.0	23.9–32.6

Source: NHPI-NHIS 2014

^a^Weighted means and percentages. Valid estimates do not account for missing data

Note: FPL: Federal Poverty Level. CD: Chronic Diseases. GED: General Educational Development

ED/ER: Emergency department/Emergency room. US: United States

**Table 2:** Predicting use of ED services among NHPIs: Odds-Ratio and Confidence Interval (95% CI)^a^

	Model 1: Predisposing, Enabling, and Evaluated Needs				Model 2: Predisposing, Enabling, and Perceived Needs		
Predisposing factors	Single vs. Non-Users	Multiple vs. Non-Users	Multiple vs. Single		Single vs. Non-Users	Multiple vs. Non-Users	Multiple vs. Single
Age	1.007 (0.997–1.018)	1.010 (0.993–1.027)	1.002 (0.993–1.011)		1.008 (0.997–1.020)	1.015* (1.001–1.030)	1.007 (0.998–1.015)
Females	0.861 (0.627–1.181)	0.823 (0.538–1.261)	0.957 (0.801–1.143)		0.914 (0.707–1.182)	0.852 (0.526–1.383)	0.933 (0.731–1.191)
High-school or less	1.083 (0.564–2.081)	1.109 (0.487–2.526)	1.024 (0.851–1.232)		1.088 (0.621–1.906)	1.161 (0.451–2.988)	1.067 (0.721–1.581)
High-school/GED	1.208 (0.795–1.835)	1.277 (0.815–2.000)	1.057 (0.901–1.240)		1.061 (0.721–1.563)	1.111 (0.590–2.092)	1.047 (0.815–1.345)
Married/living with partner	0.721 (0.423–1.228)	0.655 (0.333–1.288)	0.908 (0.648–1.273)		0.757 (0.441–1.298)	0.609 (0.279–1.329)	0.806 (0.566–1.148)
Unemployed	1.002 (0.652–1.540)	1.003 (0.575–1.748)	1.001 (0.882–1.135)		0.951 (0.694–1.304)	0.915 (0.518–1.616)	0.962 (0.742–1.246)
Born in the US	1.302 (0.841–2.014)	1.407 (0.810–2.445)	1.081 (0.823–1.420)		1.184 (0.793–1.767)	1.349 (0.703–2.590)	1.140 (0.841–1.544)
Enabling factors							
< 100% FPL	1.387 (0.705–2.731)	1.528 (0.563–4.148)	1.101 (0.716–1.695)		1.158 (0.620–2.164)	1.297 (0.391–4.304)	1.120 (0.621–2.022)
100–199% FPL	1.433 (0.875–2.348)	1.593* (1.023–2.482)	1.112 (0.829–1.492)		1.128 (0.738–1.726)	1.239 (0.633–2.427)	1.098 (0.838–1.440)
200–399% FPL	1.003 (0.656–1.533)	1.004 (0.580–1.738)	1.001 (0.883–1.134)		0.925 (0.645–1.328)	0.871 (0.460–1.650)	0.942 (0.708–1.254)
Uninsured	0.920 (0.263–3.224)	0.898 (0.166–4.850)	0.976 (0.631–1.509)		0.936 (0.369–2.379)	0.890 (0.159–4.982)	0.950 (0.431–2.097)
Usual source of care	1.102 (0.425–2.859)	1.134 (0.315–4.085)	1.029 (0.734–1.443)		1.086 (0.464–2.538)	1.157 (0.251–5.329)	1.066 (0.538–2.110)
Use eHealth information	1.520 (0.917–2.519)	1.719* (1.080–2.736)	1.131 (0.792–1.616)		1.436 (0.912–2.262)	1.901* (1.137–3.180)	1.117 (0.873–1.430)
Ability to afford health care	1.236 (0.990–1.543)	1.316 (0.823–2.105)	1.064 (0.809–1.400)		1.154 (0.980–1.359)	1.290 (0.893–1.864)	1.12 (0.873–1.437)
Neighborhood social support	0.951 (0.884–1.023)	0.937* (0.881–0.997)	0.985 (0.948–1.025)		0.971 (0.914–1.031)	0.948 (0.877–1.026)	0.977 (0.949–1.006)
Barriers to health care access	1.138 (0.809–1.601)	1.183 (0.797–1.756)	1.039 (0.916–1.179)		1.151 (0.837–1.582)	1.283 (0.804–2.048)	1.115 (0.916–1.357)
Evaluated needs (CDs)				Perceived needs (health status)			
1	1.191 (0.763–1.860)	1.254 (0.715–2.199)	1.053 (0.862–1.286)	Good	0.484* (0.282–0.830)	0.275** (0.107–0.708)	0.570 (0.241–1.347)
≥ 2	1.881 (0.971–3.645)	2.267** (1.399–3.672)	1.205 (0.714–2.034)	Very good	0.369** (0.202–0.674)	0.170*** (0.074–0.391)	0.462 (0.161–1.324)
				Excellent	0.451** (0.254–0.801)	0.243** (0.090–0.659)	0.540 (0.212–1.374)

Source: NHPI-NHIS 2014

^a^Referent: More than high-school; other marital status (widowed, divorced or separated, never married); ≥400% FPL; insured; no usual source of care; did not use eHealth; No CD; poor-fair health)

Note: FPL: Federal Poverty Level. CD: Chronic Diseases. GED: General Educational Development

ED/ER: Emergency department/Emergency room. US: United States

*p < 0.05, **p < 0.01, ***p < 0.001

Table 1 - The numbers in the first row labeled ‘Non-Users’ should read “81.6” and “79.1–83.9” instead of “816” and “791–839”.

Table 2 - In the row labeled “High school/GED” under the “ Model 2: Predisposing, Enabling, and Perceived Needs ” heading, two of the three cells contain inaccurate results as mentioned below:

**Incorrect values:**

Single vs. Non-Users:

- 1.088
- (0.621–1.906)

Multiple vs. Single:

- 1.067
- (0.721–1.581)

**Correct values:**

Single vs. Non-Users:

- 1.061
- (0.721–1.563)

Multiple vs. Single:

- 1.047
- (0.815–1.345)
